# The Rare, Unexpected Condition of a Twisted Leiomyoma in Mayer-Rokitansky-Küster-Hauser (MRKH) Syndrome: Etiopathogenesis, Diagnosis and Management. Our Experience and Narrative Review of the Literature

**DOI:** 10.3390/ijerph18115895

**Published:** 2021-05-31

**Authors:** Federico Romano, Stefania Carlucci, Guglielmo Stabile, Giuseppe Mirenda, Mariateresa Mirandola, Francesco Paolo Mangino, Andrea Romano, Giuseppe Ricci

**Affiliations:** 1 Institute for Maternal and Child Health, I.R.C.C.S. “Burlo Garofolo”, 34100 Trieste, Italy; federico.romano@burlo.trieste.it (F.R.); francesco.mangino@burlo.trieste.it (F.P.M.); giuseppe.ricci@burlo.trieste.it (G.R.); 2 Department of Obstetrics and Gynecology, Azienda Sanitaria Universitaria Giuliano-Isontina, San Polo Hospital, Gorizia-Monfalcone, 34127 Trieste, Italy; s.carlucci86@gmail.com; 3 Clinical Department of Medical, Surgical and Health Sciences, University of Trieste, 34137 Trieste, Italy; giuseppe.mirenda@burlo.trieste.it (G.M.); tea.mirandola@gmail.com (M.M.); 4 UCO Pathological Anatomy and Histology, Azienda Ospedaliera-Universitaria Ospedali Riuniti, 34129 Trieste, Italy; andrea.romano@asuits.sanita.fvg.it

**Keywords:** Mayer-Rokitansky-Küster-Hauser, leiomyoma, pelvic mass, torsion

## Abstract

Uterine leiomyomas are a common finding in medical practice, but their frequency changes drastically when contextualized in a syndrome, as in the following case. A 50-year-old woman with a known Mayer-Rokitansky-Küster-Hauser (MRKH) syndrome presented at our clinic with abdominal pain located in the lower quadrants and scarcely responsive to analgesic therapy. A twisted gynecological pelvic mass was diagnosed, and management for prompt resolution was adopted. Histologically the mass was described as a leiomyoma. The aim of the present study is to share our experience and to review the literature to compare different manifestation of the disease and different approach used in the various centers. The additional novelty of the paper is the immunohistochemical study we carried out on the leiomyoma that is contrasted with the current etiopathogenetic theories.

## 1. Introduction

Mayer-Rokitansky-Küster-Hauser (MRKH) syndrome is one of the most common causes of primary amenorrhea, second only to gonadic dysgenesis, having a frequency of 1:4000–5000 women [1]. This syndrome manifests as an aplasia or hypoplasia of the uterus and the upper two-thirds of the vagina, regular ovaries and tubes and a normal development of secondary sexual characteristics [2]. The karyotype is generally normal (46, XX) and the manifestation is secondary to an abnormal development of the müllerian ducts.

The syndrome has three different forms: type 1, typical form, is characterized by congenital absence of the uterus and the upper two-thirds of the vagina with regular adnexa; type 2, atypical form, manifests alterations to the genital apparatus (ovaries) and renal defects; type 3 shows aplasia or hypoplasia of the uterus and vagina in association with renal malformations (ectopic kidney, renal agenesis, horseshoe kidney) as well as skeleton, cardiac, ocular and hearing alterations, known as MURC syndrome [3]. In the past, the syndrome has been considered as a sporadic anomaly, but the increasing number of familial cases supports the hypothesis of a genetic cause. In these cases, the syndrome appears to be transmitted as an autosomal dominant trait with incomplete penetrance and variable expressivity. However, the etiology of MRKH syndrome remains unclear. 

The presence of leiomyomas in patients with MRKH syndrome is rare and, according to a recent review, only one other case has been described in Italy [4]. The majority of leiomyomas develop in women with a mono or bilateral rudimental uterus; in the literature, a leiomyoma in the absence of a rudimental uterus is described only in two cases. Diagnostic imaging may not give an unequivocal interpretation. In some cases, CT scans, as well as MRI, are not accurate in the diagnosis of leiomyoma or its anatomical delineation [5,6]. Therefore, in most cases, surgery is the only diagnostic tool to define the nature of the lesion.

Leiomyomas are benign tumors derived from smooth muscle cells forming the myometrium. They represent the most common benign female genital neoplasia with an estimated frequency of 20% of the population between 30- and 60-year-old women [7]. In the current study, we would like to describe the case of a 50 year old patient with MRKH syndrome who was hospitalized with acute abdomen due to intraoperative evidence of a para-ovarian pelvic neoformation twisted on its own peduncles. Furthermore, the purpose of this study is to identify the number of cases of leyomyomas in patients with MRKH reported in the literature and how many of these manifested as an acute abdomen. Secondly, in these selective cases, we wanted to evaluate and compare the surgical approaches adopted. In addition, we analyzed the immunohistochemical aspect of the leiomyoma.

## 2. Case

A 50-year-old woman presented in the emergency room who had been experiencing abdominal pain for one week. Initially the pain was spread throughout the abdomen and was similar to cramps; later on, it started to be confined and localized in the abdominal inferior quadrants and was scarcely responsive to an analgesic therapy. There were no accompanying symptoms or increased inflammatory markers. The patient was aware of being affected by MRKH syndrome, which was diagnosed in puberty during a follow-up for a primary amenorrhea. The patient was also aware of a pelvic mass of unknown origin discovered 5 years prior during a routine ultrasound. During the anamnesis, the patient denied having menstrual flows and pelvic pains in the past. 

At the medical examination, the abdomen was soft and non-tender except for a strong tenderness corresponding to a hardened hypogastric tumefaction. The Blumberg sign was negative. Blood tests were requested and came out normal. White blood count and C reactive protein were negative. An abdominal ultrasound was performed as well as a computed tomography, with identification of both kidneys, normal in size and position, with smooth profiles and a regular echogenicity and parenchymal thickness, without any signs of cystic alterations in their context. There was no sign of hydronephrosis on the right side; mild pyelectasis was found on the left kidney. The bladder was expanded with regular walls. A neoformation of unknown origin was identified in the pelvic region described as a voluminous pelvic expansive formation (major transverse diameter: 9 cm; antero-posterior diameter: 11 cm) likely referred to a voluminous uterine partially calcified leiomyoma. Mild perihepatic and perisplenic effusion were also detected; liver, pancreas, spleen, adrenal glands and kidneys were normal. 

At the gynecologic examination, the vagina was atresic, and a hard hypomobile tumefaction was identified in the pelvic region with its extension reaching 2 cm above the transverse umbilical line.

**Figure 1:**
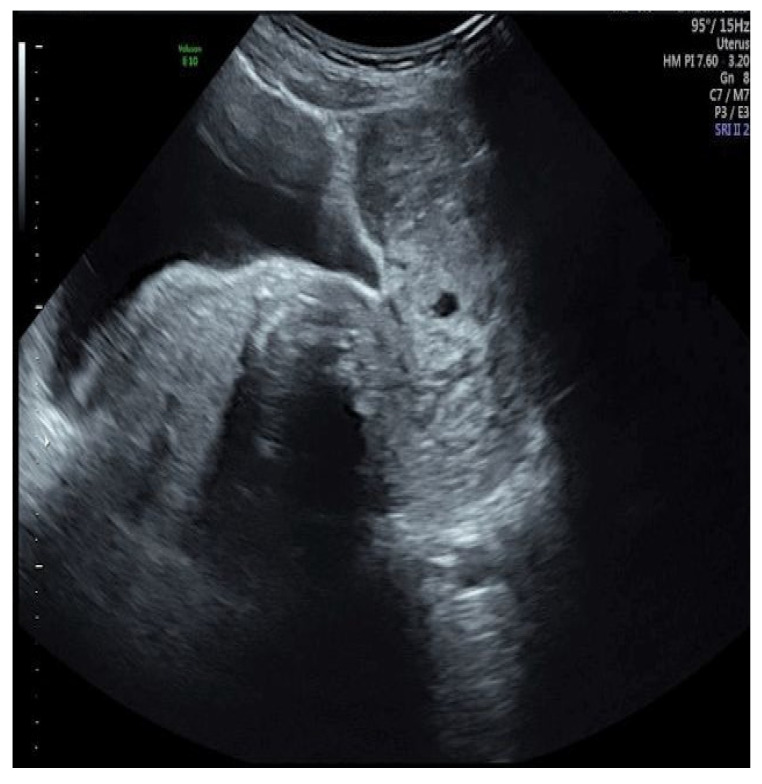
Ultrasound image of the lesion.

At transrectal and transvaginal ultrasonography examination (Voluson GE-E10; TV probe 5–9 MHz and TA probe 1–5 MHz), the uterus was not recognizable nor was the endometrium visible, and the whole pelvis was occupied by a 15 cm diameter solid bilobate “C-shape” well-defined lesion (Figure 1) with mixed echogenicity as listed below: intense internal shadows with calcifications, material of ground-glass appearance surrounded by a hyperechogenic rim, peripheral moderate vascularization (color score 2) with no translesional vascularity, as described by MUSA terminology [8].

In addition, we described a ureterocele image in the left kidney, possibly due to extrinsic compression by the pelvic malformation or by primary renal malformation, as described in MRKH disease. Moderate peritoneal effusion was present in the pelvis.

**Figure 2:**
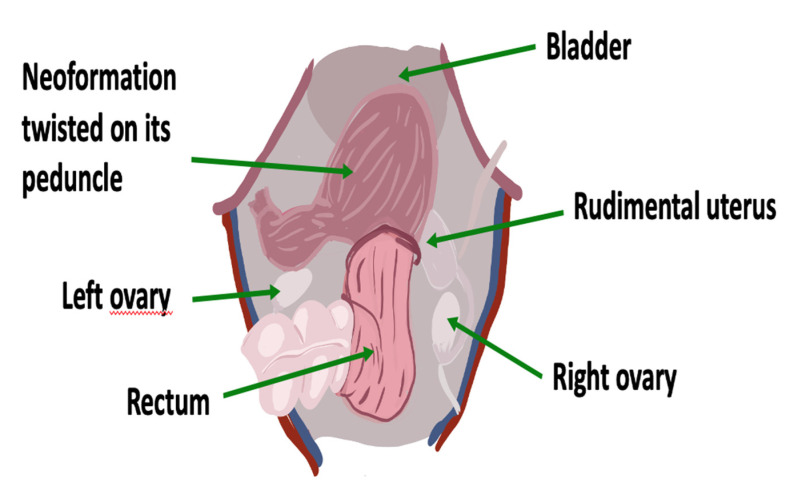
Anatomical relationships of the rudimentary uterus and of the neoformation inside the pelvis.

Due to the worsening of the pain and no response to the analgesic therapy, a surgical approach was chosen, with a supposed diagnosis of an unknown origin pelvic lesion in a patient with MRKH syndrome. An initial laparoscopic approach was undertaken with the insertion of an umbilical 10 mm optical trocar and three 5 mm ancillary trocars. A rudimental uterus was highlighted on the right side of the pelvis with bilateral regular ovaries. A 15-cm dark-red left formation was found, departing from the rudimental uterus and twisted on its peduncle (Figure 2). 

Considering the size and hardness of the mass due to calcifications present in its context, as well as the uncertain histologic nature of the mass, we decided to convert the surgery from laparoscopy to laparotomy by a transverse suprapubic mini-laparotomy. A small quantity of peritoneal liquid was drained and sent for cytological evaluation. After identification of the left ureter by retroperitoneal access, we proceeded with the removal of the entire mass with its vascular peduncle. 

**Figure 3:**
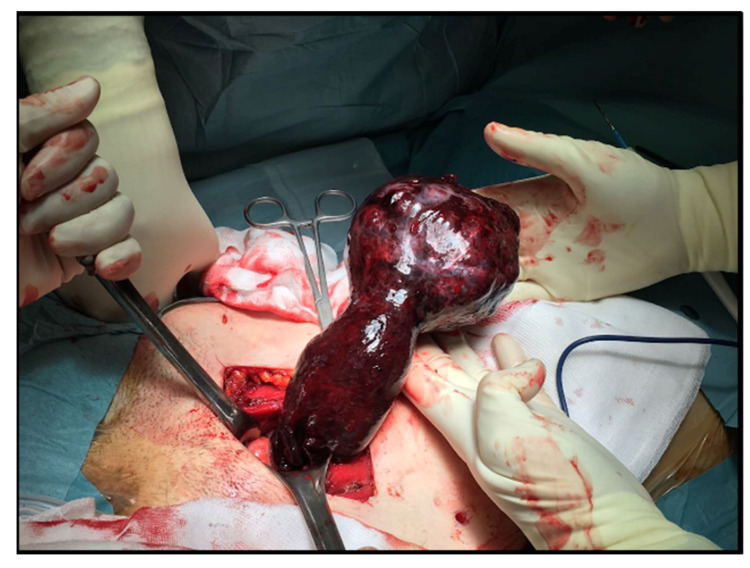
Leiomyoma twisted on its peduncle outside the abdomen.

**Figure 4:**
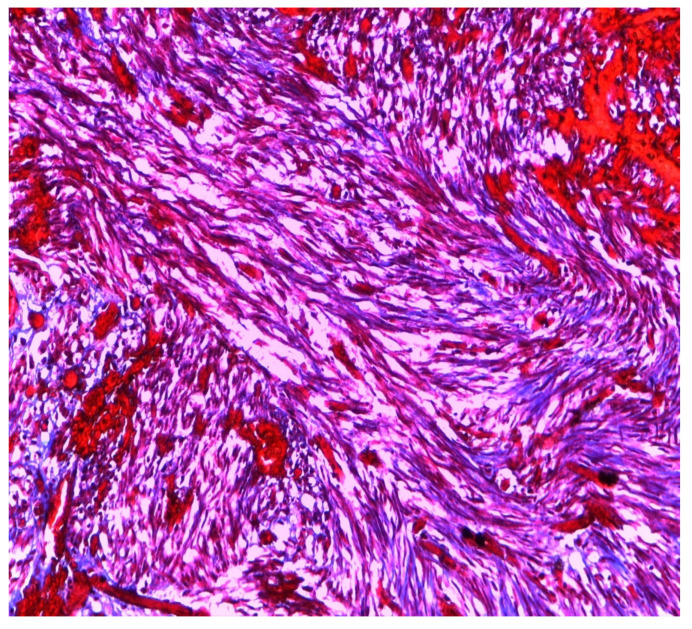
Histological image of leiomyoma stained with Trichrome-Masson.

**Figure 5:**
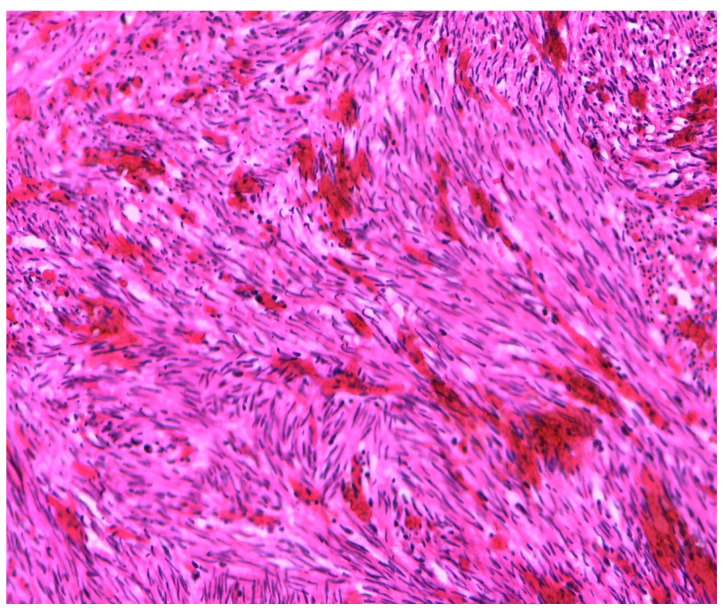
Histological image of leiomyoma stained with hematoxylin.

**Figure 6:**
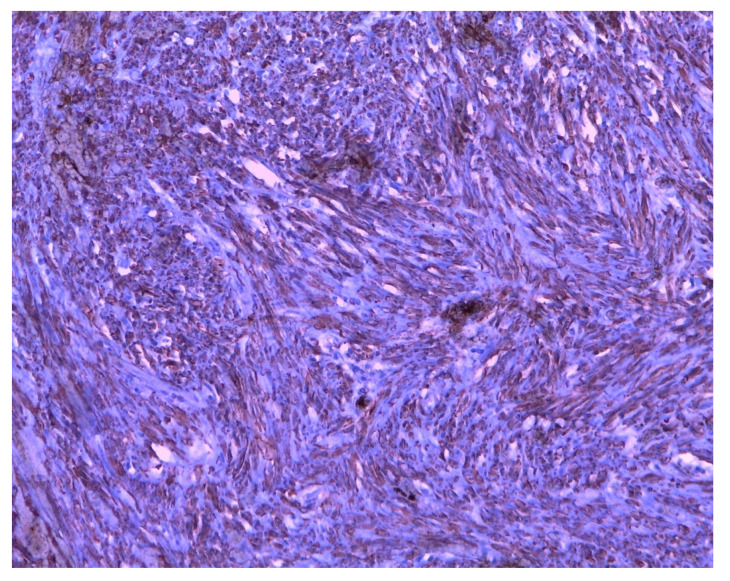
Histological image of leiomyoma with smooth muscle actin stain.

The mass (Figure 3) was, therefore, sent for histological evaluation. The diagnosis was a spindle-cell neoplasia, partially ossified, with sclero-hyaline areas characterized by hemorrhagic centers and a lumpy and congested area, measuring 10 cm × 9 cm × 7 cm and weighing 460 g with a fibroadipose congested extension measuring 10 cm × 5 cm × 3 cm; the morphologic and immunohistochemical findings are coherent with hemorrhagic sclero-hyaline leiomyoma, possibly caused by a torsion (Figure 4, Figure 5 and Figure 6). Moreover, the immunohistochemical analysis was negative for the presence of progestin and estrogenic receptors.

## 3. Materials and Methods

A search was carried out on PubMed and Scopus (until December 2020) to identify articles involving patients with MRKH and leiomyomatosis. The manuscripts considered were published from 1999. The research was focused on original articles in English. Nine manuscripts were detected through the references of the works that had been identified with the research on PubMed and Scopus. A total of 35 cases were found describing the presence of myomatosis in patients with MRKH where 27 were not complicated and 8, instead, had an acute manifestation similar to our case.

## 4. Results

Apparently, there are only 27 cases described of uterine leiomyoma in MRKH not complicated by acute abdomen (Table 1) [2,3,4,9,10,11,12,13,14,15,16,17,18,19,20,21,22,23,24,25,26,27,28,29,30,31]. The average age at the time of diagnosis was 41.8 years. The cases described have been evaluated for patients undergoing routine examination or urgent assessment due to chronic symptomatology, not for acute pain, as in our case. The preferred surgical approach is laparotomy; in only four cases the mass has been removed in laparoscopy. In almost all cases, the lesion originated from a rudimental uterine horn and apparently in only three cases, it was not.

Focusing our attention on cases with acute onset similarly to ours, we found only eight cases where a torsion of the lesion was described [5,6,31,32,33,34,35,36,37]. In six cases, the clinical manifestation was an acute abdomen and, apparently, only in two cases, the torsion concerned only the mass and not the annexes.

To better understand the manifestation of the disease and the different management of it worldwide, we proceeded to analyze a few features, as follows. In almost all cases, the first suspicion was given by ultrasound since it is the faster instrument used in the emergency room. After that, magnetic resonance and computed tomography were used to confirm the diagnosis. 

The average age of onset of the disease was 45.5 years old, ranging from 28 to 55 years old and in line with the typical age of leiomyoma manifestation. The volume of the masses is similar in almost all cases, around 10 cm × 15 cm. Only in one case, the dimension of the pelvic mass was smaller, and in two studies, there is no mention of the volume of the myomas. The mass origin was from a rudimentary uterus in four cases, paraovarian or adnexal in three cases and not clarified in the remanent two cases. Regarding the surgical approach, all the surgeons preferred laparotomy access with respect to a laparoscopic one, with the only exception of Yi et al. [34] who, unfortunately, did not report the dimension of the lesion; therefore, an analysis of their choice is not possible. Finally, four out of nine surgeons removed the twisted mass only, whereas the others chose the removal of the rudimental uterus.

## 5. Discussion

The presence of a pelvic mass in a woman with MRKH can be a diagnostic dilemma due to a subverted gynecologic anatomy and the frequent association with renal malformations. The low incidence of the disease makes the diagnosis even more difficult. Our experience and a review of the literature demonstrate that leiomyomas may develop in the context of a rudimental uterus, but it is a rare finding. Specifically, our case falls within the classic form of the syndrome, with the presence of a rudimental unilateral uterus. Clinically, it seems that the mass can be completely asymptomatic until its volume reaches great dimensions (10–15 cm). Larger or smaller myomas, generally, do not lead to an acute complication. This is probably due to the poor mobility and, therefore, to the reduced possibility of torsion of myomas of these sizes.

The age of onset is the same of the population with normal gynecologic development, and surgeons almost unanimously prefer the laparotomic approach in emergency cases such as the ones analyzed in the current review.

On ultrasound examination, leiomyomas are hypoechoic or heterogeneous masses. Sometimes there can be an internal cystic degeneration with necrosis or hemorrhage that appears ultrasonographically as a cystic component with internal echogenic material; differently, calcifications appear as hyperechoic foci [22]. 

The pre-surgical differential diagnosis between an ovarian lesion and a lesion of a rudimental uterus may be difficult since the usual anatomical landmarks are subverted. For example, a large pelvic mass with possible inner myxomatous degeneration may be easily confused with a cystic ovarian lesion [11].

If the mass is associated with pelvic pain, adenomyosis and other pathologies should be considered in the differential diagnosis, as described in rare cases [35,38]. In our case, the patient denied a history of pelvic pain, endometriosis and she declared that she never had a menstrual cycle. This excluded from the differential diagnosis the possibility of hematocolpos or pain related to the hormonal cycle.

Among the few cases described in the literature, apparently only eight are reported to be the manifestation of the lesion as a twisted mass, and six patients had an acute abdomen. Furthermore, only in two reports, the torsion involved the mass exclusively, sparing the adnexa [5,6], similarly to what happened in our case (Table 2), where both surgical procedure and histological examination highlighted that neither the uterus or ipsilateral Fallopian tube were involved in the torsion. This allowed us to save the ovaries, preserving the hormonal balance of the patient. Apparently, this is the second case in which it was possible to spare the patient’s ovaries.

Regarding the surgical procedure, the literature describes various approaches. Laparoscopic, as well as laparotomic techniques, have been chosen according to the patient’s clinical evaluation, imaging, number of masses, mass localization and characteristics (size of the mass or complicated nature of its feeding arteries). Removing the remnant uterus may be a choice in order to prevent the risk of recurrence [21,32] and a mono or bilateral salpingo-oophorectomy may be added to the procedure, according to the mass location or complication [33]. In conclusion, even if our case seems to be clinically similar to a few others described in the literature, in the current paper, we would like to share a particular immunohistochemical aspect we have discovered.

**Figure 7:**
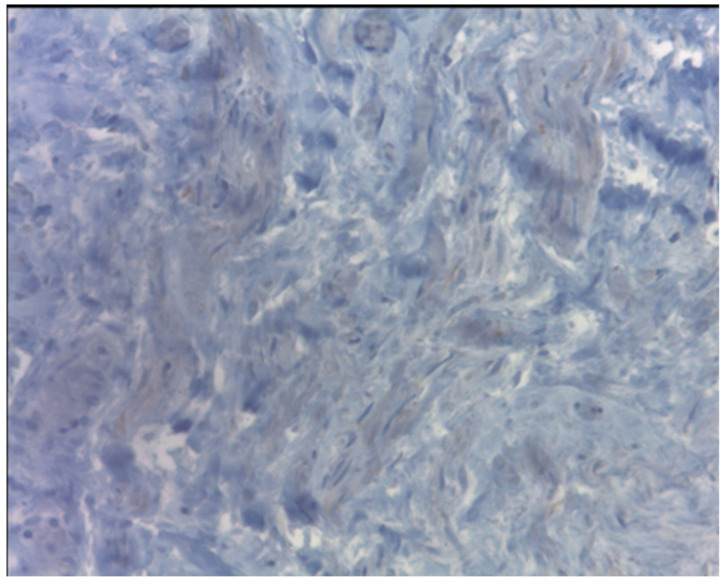
In the tissue, the presence of estrogenic receptors was negative.

**Figure 8:**
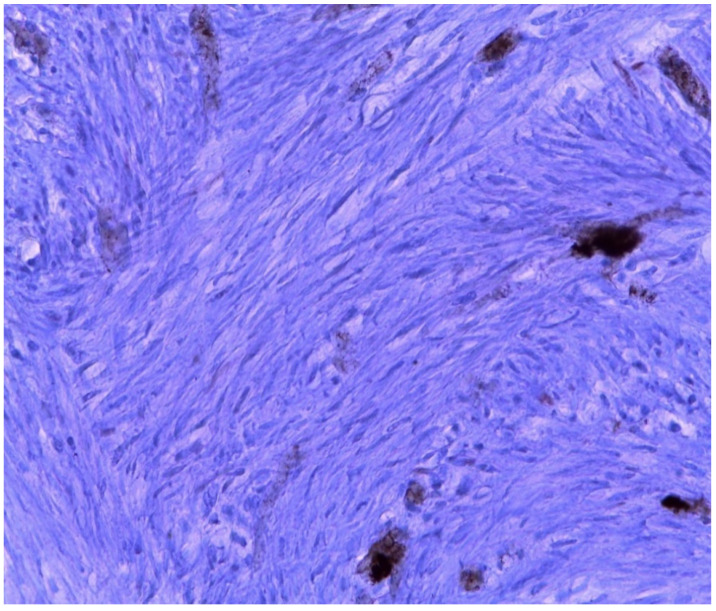
In the tissue, the presence of progestin receptors was negative.

In our review, only one case is described as a mitotically active leiomyoma [23], and the etiopathogenesis remains unclear. The current knowledge about it suggests that the pathogenesis of fibroids is a multistep process, starting with the recruitment of a smooth muscle stem cell from the myometrium that lacks receptors for the gonadal steroids. However, under the influence of specific driver mutations, in addition to estrogen, progesterone and WNT–β-catenin signaling, the stem cell differentiates into a preclinical fibroid. Subsequently, four key cell types that comprise fibroids (smooth muscle cells, vascular smooth muscle cells, fibroblasts and fibroid-associated fibroblasts) and the extracellular matrix (ECM) synergize with environmental and molecular stimuli to undergo growth acceleration and progression into clinical disease [39,40]. Therefore, the development of fibroids in an MRKH patient can be explained by the presence of smooth muscle cells in the proximal ends of the müllerian ducts, stimulated by the estrogen physiologically produced by the ovaries, resulting in the formation of leiomyomas; in these cells, the estrogenic receptors seem to be overexpressed [3]. However, Sharma et al. attribute low concentration or sensibility to the estrogenic stimulation by the receptors located on the rudimental uterine horns in women with MRKH [10]. Unfortunately, due to the worsening pain and urgent surgery, it was not possible to administer a perioperative hormonal dosage to our patient. However, a receptors study conducted over the mass removed from the patient presented quite a curios result. The presence of estrogenic and progestin receptors was negative (Figure 7 and Figure 8). Therefore, our finding, in contrast with what some authors demonstrated in the past [41], reinforces the doubts about the etiopathogenesis of fibroids both in MRKH patients and in the general population. A potential bias in this case could be the ischemic condition caused by the torsion of the mass that could lead to a partial alteration of the tissues. 

## 6. Conclusions

In women with Mayer-Rokitansky-Küster-Hauser syndrome presenting with pelvic pain not responsive to the analgesic therapy, the presence of a gynecological complication should always be considered among the possible diagnosis. The use of ultrasonography is recommended, adding a CT or an MRI for higher anatomical detail and better pre-surgical stadiation. However, the proper and final diagnosis, due to the rarity and the anatomical complexity of these lesions, can be given only through surgical procedure and histological evaluation. 

We demonstrated that the etiopathogenesis of leiomyomas still remains unclear, and the cluster of agonists and antagonists with their receptors involved in the development needs to be clarified. 

Finally, from our review, it emerges that the occurrence of new myomatous neoplasms in patients with MRKH, starting or not from uterine rudiments, could cause an acute abdomen requiring urgent surgery. For this reason, patients who undergo pelvic surgery could benefit from preventive remotion of uterine residues by minimally invasive surgery with minimal discomfort to the patients.

## Figures and Tables

**Table 1:** Cases of uterine leiomyomas in Mayer-Rokitansky-Küster-Hauser (MRKH) syndrome.

MRKH Syndrome and Myomatosis								
Case	Authors	Nationality	Age of Patients	Symptoms	Imaging	Location	Dimensions	Tipe of Surgery
1	Rhee Abdom. Imaging 1999 [27]	Korea	49	None	MRI	Rudimentary uterus	10 cm × 10 cm × 6 cm	LPS
2	Tsin J. Am. Assoc. Gynecol. Laparosc. 2000 [28]	USA	36	None	US + CT	-	8.4 cm	LPS
3	Endemonds J. Pediatr. Adolesc. Gynecol. 2003 [17]	England	70	Abdominal swelling –nocturia	-	-	10 cm	LPT
4	Jadoul Fertil. Steril. 2004 [15]	Belgium	42	Dyspareunia	US-MRI	Left rudimentary uterus	10 cm	LPS
5	Deligeoroglu Fertil. Steril. 2004 [20]	Greece	Case I 42 Case II 38	Case I: chronic pain Case II: None	US	Rudimentary uterus	Case I: 5.9 cm × 5.5 cm Case II: 5.8 cm × 9.6 cm	LPS
6	Papa Fertil. Steril. 2008 [4]	Italy	47	Chronic pain	US + MRI	Retrovescical space	15 cm	LPS
7	Lamarca Fertil. Steril. 2009 [18]	Spain	35	Infertility	US-MRI	Rudimentary uterus	3–5 cm	LPT
8	Lanowska Fertil. Steril. 2009 [26]	Germany	39	None	US + MRI	Rudimentary uterus	8.5	LPS
9	Pati J. Indian Med. Assoc. 2009 [31]	India	20	Primary amenorrhea Chronic pain	-	Rudimentary uterus	8 cm × 7 cm	LPT
10	Fukuda J. Obstet. Gynaecol. Res 2010 [29]	Japan	50	Chronic pain	CT + MRI	Rudimentary uterus	7 cm	LPT
11	Chun J. Menopausal Med. 2013 [14]	Korea	55	None	MRI	Rudimentary uterus	5.4 cm × 4.8 cm × 4.7 cm	LPT
12	Pàez Lòpez Rev. Colomb. Obstet. Ginecol. 2013 [16]	Colombia	32	Chronic pain	US	Rudimentary uterus	9 cm	LPS
13	Rawat J. Radiol. Case Rep. 2013 [19]	India	35	None	US-CT-MRI	Rudimentary uterus	25 cm × 18 cm × 12 cm	LPT
14	Kulkarni J. Hum. Reprod. Sci. 2015 [21]	India	25	Primary amenorrhea	US-MRI	Rudimentary uterus	5 cm × 4 cm × 4 cm	LPT
15	Girma Ethiop. J. Health Sci. 2015 [22]	Ethiopia	40	Chronic pain	US	Right broad ligament and right Mullerian bulb	18 cm × 10 cm × 8 cm	LPT
16	Narayanan BMJ Case Rep. 2015 [11]	India	43	Chronic pain	US + MRI	Rudimentary uterus	-	LPT
17	Hasegawa Obstet. Gynecol. 2015 [13]	Japan	40	Abdominal swelling	US + MRI + CT	Rudimentary uterus	20 cm × 30 cm	LPT
18	Park J. Obstet. Gynaecol. 2016 [12]	Korea	36	None	CT	Rudimentary uterus	16 cm × 92 cm	LPT
19	Salem Facts Views Vis. Obgyn. 2016 [2]	Iraq	40	None	US	Right-sides intra-peritoneal space of round ligament	7 cm × 9 cm	LPT
20	Dimitriadis J. Reprod. Med. 2016 [23]	USA	43	None	-	-	-	LPS+LPT
21	Amaratunga Ultrasound Quarterly 2017 [3]	Canada	66	Chronic pain	US + MRI	Right annexed area	4.5 cm × 4 cm	LPS
22	Sharma Int. J. Gynaecol. Obstet. 2017 [10]	India	45	Chronic pain	US + CT	Right and left rudimentary horn	18 cm × 15 cm; 5 cm × 4 cm	LPT
23	Karthik J. Gynecol. Surg. 2017 [24]	India	33	Primary amenorrhea Dyspareunia	US-MRI	Rudimentary uterus	7.6 cm × 5.3 cm	LPS
24	Albahlol Eur. J. Obstet. Gynecol. 2019 [30]	Egypt	45	Primary amenorrhea Dyspareunia	US + MRI	-	15 cm × 12 cm	LPT
25	Jokimma BMC Women’s Health 2020 [9]	Finland	47	None	US-MRI	-	6 cm	-
26	Harzif Int. J. Surg. Case Rep. 2021 [25]	Indonesia	38	Chronic pain	US + MRI	Rudimentary uterus	6 cm 5 cm	LPS

**Table 2:** Torsion of leiomyomas in Mayer-Rokitansky-Küster-Hauser (MRKH) syndrome in the literature.

References	Age of Onset	Acute Abdomen	Surgical Approach	Imaging	Dimension of the Lesion	Mass Localization	Type of Surgery
YAN, et al. (2002) [6]	52	Yes	LPT	US + CT	15 cm	Rudimentary uterus	Hysterectomy and bilateral salpingo-oophorectomy
Galajova, et al. (2003) [37]	55	No	LPT	US	10 cm × 7.5 cm	Not reported	Mass only
Petric, et al. (2008) [36]	53	Yes	LPT	Not reported	Date not available	Not clear origin	Mass only
FLETCHER, et al. (2012) [5]	28	Yes	LPT Pfannenstiel	MRI	10 cm × 15 cm	Paraovarian mass	Mass only
VIDYASHREE, et al. (2014) [32]	40	No	LPT	CT + MRI	6 cm × 7 cm and 5 × 6 cm	Rudimental uterus	Bilateral salpingo-oophorectomy and excision of uterine remnant
KUNDU, et al. (2014) [33]	40	Yes	Vertical LPT	US + CT	10 cm	Rudimentary uterus	Right salpingo-oophorectomy, excision of right and left hemiuteri with pedunculated leiomyomas, and left salpingectomy
YI, et al. (2016) [34]	47	Yes	LPS	MRI	Date not available	Rudimentary uterus	Bilateral salpingo-oophorectomy and excision of uterine remnant
HOO, et al. (2016) [35]	45	Yes	LPT	US + MRI	15 cm × 13 cm × 13 cm	Right adnexa	Right salpingo-oophorectomy and excision of uterine remnant
Case Described by This Article	50	Yes	LPT Pfannenstiel	US + CT	10 cm × 9 cm × 7 cm	Paraovarian mass	Mass only

## Data Availability

The authors confirm that the data supporting the findings of this study are available within the article.
